# CD45 and CD148 Are Critically Involved in Neutrophil Recruitment and Function During Inflammatory Arthritis in Mice

**DOI:** 10.3390/cells14151169

**Published:** 2025-07-29

**Authors:** Jan-Niklas Heming, Andreas Margraf, Karolina Najder, Giulia Germena, Mathis Richter, Anika Cappenberg, Katharina Henke, Bernadette Bardel, Lena Schemmelmann, Marina Oguama, Pia Lindental, Wida Amini, Jacqueline Sobocik, Georg Schett, Gerhard Krönke, Helena Block, Jan Rossaint, Oliver Soehnlein, Alexander Zarbock

**Affiliations:** 1 Department of Anesthesiology, Intensive Care and Pain Medicine, University Hospital Münster, 48149 Münster, Germany; 2 The William Harvey Research Institute, Barts and The London School of Medicine and Dentistry, Queen Mary University of London, London E1 2AD, UK; 3 Institute of Experimental Pathology (ExPat), Centre of Molecular Biology of Inflammation (ZMBE), University of Münster, 48149 Münster, Germany; 4 Department of Internal Medicine 3—Rheumatology and Immunology, Friedrich-Alexander-University Erlangen-Nürnberg and Universitätsklinikum Erlangen, 91054 Erlangen, Germany; 5 Department of Rheumatology and Clinical Immunology, Charité-Universitätsmedizin Berlin, 10117 Berlin, Germany; 6 Department of Anesthesiology, Critical Care and Pain Medicine, McGovern Medical School, The University of Texas Health Science Center at Houston, Houston, TX 77030, USA

**Keywords:** arthritis, neutrophils, neutrophil recruitment, integrins, Src family kinases

## Abstract

Neutrophils play a key role in autoimmune diseases like rheumatoid arthritis, contributing to tissue damage through rapid recruitment and activation. In this study, we investigated the regulatory properties of two receptor-like tyrosine phosphatases (RPTPs), CD45 and CD148, in inflammatory arthritis. Using an in vivo mouse model of K/BxN serum transfer-induced arthritis, we found that CD45 and CD148 feature distinct regulatory properties during inflammatory arthritis. CD45 is required for neutrophil infiltration, cytokine release, and reactive oxygen species production, whereas CD148 deficiency leads to a delayed onset of arthritis but unaltered overall neutrophil infiltration and reduced ROS production. Furthermore, we could demonstrate that activation of Src family kinases in neutrophils is differentially regulated by CD45 and CD148 in a stimulus-dependent manner. Summarizing, our results suggest that CD45 is positively involved, while CD148 is positively and negatively involved in neutrophil recruitment and function during inflammatory arthritis.

## 1. Introduction

Rheumatoid arthritis (RA) is an autoimmune systemic disorder characterized by inflammatory arthritis. The prevalence of RA is around 0.5–1% worldwide, placing a substantial burden on healthcare systems [1]. In inflamed joints, neutrophils are among the most frequent leukocyte subsets and contribute to tissue damage by releasing reactive oxygen species (ROS), neutrophil extracellular traps (NETs), and cytokines that activate other immune cells, thereby exacerbating inflammation as well as tissue damage [2]. Therefore, a better understanding of neutrophil recruitment and functionality in the context of inflammatory arthritis is needed.

The experimental arthritis model induced by K/BxN serum transfer (serum transfer-induced arthritis, STIA) imitates important aspects of human RA. These include immune complex (IC) formation in the joint, synovitis, and cartilage damage, mainly dependent on the innate immune system, whereas B- and T-cells are dispensable [3,4,5,6]. Therefore, neutrophil recruitment as well as effector functions are crucial for the development of arthritis in the K/BxN mouse model, similar to joint inflammation in RA [3,6,7,8,9]. Indeed, the onset of the clinical disease is characterized both in patients and in the K/BxN model by neutrophils being recruited into the joint [2,7].

Src family kinases (SFKs) are essential regulators of leukocyte functions, including integrin activation, Fc-receptor signaling, and ROS production [5,10,11,12,13,14]. Since these processes need to be precisely regulated, the activation status of the SFKs themselves is closely controlled by two regulatory tyrosine sites [15,16]. Activation requires phosphorylation of a conserved tyrosine in the activation loop, hereafter referred to as tyrosine 416 (Y416, according to mammalian Src; position of analogue residue varies among species and SFK [15,16,17,18,19]). This phosphorylation leads to the highly active state by conformational changes that allow substrate binding and the catalytic cleft to form a kinase-competent structure [16,20]. Inhibition of SFK activity is induced by phosphorylation of the C-terminal regulatory site, subsequently named tyrosine 529 (Y529, related to mammalian Src; position of analogue residue changes between species and SFK [15,16,17,18,19]). This generates an inactive configuration through positioning of the dephosphorylated activation loop in its inactive position, thereby preventing the formation of a kinase-competent structure [15,16,21].

The receptor-like protein tyrosine phosphatases (RPTPs) CD45 (encoded by Ptprc) and CD148 (encoded by Ptprj) both regulate SFK activity through dephosphorylation of the inhibitory C-terminal tyrosine 529 [22,23,24,25]. CD45 is a type 1 glycoprotein with two receptor-type phosphatase (PTP) domains, D1 and D2, abundantly expressed in hematopoietic cells except for erythrocytes and platelets [26,27]. While the PTP domain D1 is enzymatically active, the D2 domain seems inactive. Nonetheless, both domains are required for optimal phosphatase function in vivo [26,27] as, potentially, the D2 domain may play a role in substrate access and localization. CD45 is thought to be the main regulatory phosphatase that dephosphorylates Y529 and subsequently increases SFK activity [22,24,26]. CD45-deficient leukocytes were shown to feature inactivated SFKs and reduced migration capacity in a *Staphylococcus aureus* (*S. aureus*) infection model [24]. However, there are also reports demonstrating that CD45 can be a negative regulator by dephosphorylation of SFKs at Y416 and JAK kinases [16,28,29]. Compared to CD45, the RPTP CD148 has only one PTP domain and is expressed in most hematopoietic cells as well as fibroblasts, epithelial, and endothelial cells [30,31,32]. In B-cells and macrophages, CD148 seems to have redundant functions with CD45 to positively regulate SFK activity [23]. In contrast, in neutrophils, CD148 seems to have both positive as well as negative functional regulatory effects. In a *S. aureus* infection model, neutrophil recruitment was increased, whereas phagocytosis was decreased in CD148-deficient neutrophils, suggesting that CD45 and CD148 regulate signaling pathways in distinct ways [24]. However, the RPTP-dependent intricate regulation of neutrophil recruitment and function, as well as the regulation of SFKs in the context of non-infectious, autoimmune diseases, such as rheumatoid arthritis, are still unknown.

To investigate the role of CD45 and CD148 in the onset and progression of arthritis, we used the STIA model in CD45KO (CD45 knockout, Ptprc-/-), CD148KO (CD148 knockout, Ptprj-/-), and DKO (double knockout, Ptprc-/-Ptprj-/-) mice. We additionally investigated the effects induced by the absence of CD45 and CD148 on neutrophil recruitment and activation in vivo and in vitro, and analyzed ROS production and cytokine release in vitro. We found that CD45 and CD148 are critically, but only partially redundantly, involved in the regulation of neutrophil functionality during arthritis development, affecting recruitment, cytokine release, and ROS production. Our data also suggest distinct functions of CD45 and CD148 in the activation of SFKs and SFK-related pathways in a stimulus-dependent manner.

## 2. Materials and Methods

A resources table (Supplemental Table S1) is provided with information on reagents and resources required to reproduce results presented in the manuscript.

### 2.1. Animal Models

Generation of Ptprc-/-, Ptprj-/-, and Ptprc-/-Ptprj-/- mice was described previously [23]. Animals were maintained in a specific pathogen-free facility at the University of Münster. Wild-type (WT) C57BL/6J and 8–16-week-old Ptprc-/-, Ptprj-/-, Ptprc-/-Ptprj-/- mice were used for all experiments. The Animal Care and Use Committee of the University of Münster (Germany) and the institutional review board of North Rhine-Westphalia (Germany) approved all animal experiments (LANUV NRW, animal protocol number G23.A065, date of approval: 28 June 2023; animal protocol number: 84-02.04.2016.A438, date of approval: 14 March 2017; TSB University of Münster: animal protocol number: T23.058, date of approval: 16 August 2023).

### 2.2. Serum Transfer, Measurement of Ankle Thickness, and Arthritis Scoring

Pooled serum from arthritic K/BxN mice (200 μL) was transferred into recipient mice intraperitoneally on day 0. Ankle swelling was measured with a digital thickness gauge (Hedue, Mönchengladbach, Germany). A clinical score was determined for each paw using the following scoring system: 0, normal, no visible differences compared to healthy mice; 1, one or two digits inflamed and swollen, no visible swelling of the paw or ankle; 2, three or more digits inflamed and swollen, but no paw swelling or only slight swelling of the whole paw; 3, swelling of the whole paw; and 4, severe swelling of the entire paw and all digits or ankylosed paws and digits, the mice cannot grip the top of the cage.

### 2.3. Chimeric Mice

Chimeric mice were generated as described previously [33]. Destruction of the native bone marrow (BM) of male WT recipient mice was performed by fractional lethal irradiation of 9 gray. WT and Ptprc-/-, Ptprj-/-, and Ptprc-/-Ptprj-/- mice served as donors. Approximately 5 × 10^6^ cells were injected intravenously into irradiated recipient mice. Six to eight weeks after a successful BM transplantation, K/BxN serum transfer was performed.

### 2.4. Tissue Processing of K/BxN Serum-Induced Mice

7 days after K/BxN serum injection, blood, BM, and synovial fluid (SF) were harvested for spectral flow cytometry analysis. For this purpose, mice were anesthetized by intraperitoneal administration of ketamine (100 μg/g body weight; Sanofi Winthrop Pharmaceuticals, Paris, France) and xylazine (10 μg/g body weight; Elanco, Greenfield, IN, USA), blood samples were taken by cardiac puncture, and mice were euthanized. Blood was collected, and 100 μL were directly analyzed by an automated hematology analyzer (Sysmex Deutschland GMBH, Norderstedt, Germany). Subsequently, blood was incubated with erythrocyte lysis buffer (BioLegend, San Diego, CA, USA) on ice. After washing, cells were centrifuged and resuspended in Hanks’ buffer (Hanks’ Balanced Salt Solution (HBSS) w/o Mg and Ca, 0.06% bovine serum albumin (BSA), 0.3 mM EDTA). SF samples were obtained by opening both knees against the joint and passing them over a 70 μm strainer. Cells were collected in Hanks’ buffer on ice. Afterwards, one femur was harvested for isolation of BM cells and flushed with Hanks’ buffer. Cells were incubated in erythrocyte lysis buffer (BioLegend) on ice. After washing, cells were centrifuged and resuspended in Hanks’ buffer. Additionally, hind paws were harvested for histological analyses.

### 2.5. Spectral Flow Cytometry

Samples were processed as stated above to obtain cell samples. For spectral flow cytometry, blood, BM, and SF were subjected to erythrocyte lysis and subsequently used for staining. Isolated cells from BM, blood, and SF were incubated with a mix of fluorophore-conjugated antibodies (see resources table) for 20 min on ice. After washing, flow cytometry was performed on a 5L-Cytek Aurora (Cytek Biosciences, Fremont, CA, USA). Figure S4A shows the applied gating strategy. After excluding doublets (forward and side scatter characteristics) and dead cells (DAPI), neutrophils were characterized as CD11b+Ly6CintCD115-Ly6G+. Data were analyzed using FlowJo v10 (BD Biosciences, Franklin Lakes, NJ, USA). Neutrophil counts were determined based on absolute counting beads (Thermo Fisher, Waltham, MA, USA) and volumetric counting. For analyses of neutrophil heterogeneity, neutrophils from all samples were down-sampled and concatenated. Dimensional reduction and unsupervised clustering were performed using the Uniform Manifold Approximation and Projection (UMAP) and FlowSOM plugins in FlowJo v10 (BD Biosciences). Data visualization was performed using R version 4.4.1 with RStudio (v2024.09.0+375) (including ggplot2 package).

### 2.6. Immunohistochemical Staining and Histopathological Scoring

Dissected ankles were fixed in 4% paraformaldehyde (PFA, Sigma Aldrich, St. Louis, MO, USA) for 24 h at 4 °C, demineralized in 10% ethylenediaminetetraacetic acid (EDTA, Sigma Aldrich) for 3 weeks at 4 °C, and afterwards embedded in paraffin. Sections of 4–12 mm were deparaffinized and stained with Hematoxylin/Eosin (H/E, Roth, Sigma Aldrich) and Safranin O/Fast green (Sigma Aldrich). Histopathological scoring of inflammatory infiltrates/synovitis (H/E staining, Krenn Synovitis Score) and cartilage damage (Safranin O/Fast green, Mankin Score) was performed in a blinded manner according to established scoring systems [34,35]. Exemplary images were acquired using a Lionheart FX Microscope (BioTek, Winooski, VT, USA) with 4× and 20× magnification. Image stitching and deconvolution was performed using BioTek Gen5 Software for Imaging & Microscopy (Agilent, Santa Clara, CA, USA). Analysis of paws from chimeric mice was performed using a Zeiss Axioskop microscope (Zeiss, Oberkochen, Germany) equipped with a digital camera and an OsteoMeasure image analysis system (Osteometrics, Decatur, GA, USA).

### 2.7. Immunofluorescence Staining and Fluorescence Analysis

Dissected ankles were processed as stated above. Afterwards, samples were cryopreserved with cryoprotectant solution containing sucrose (Merck, Rahway, NJ, USA) and polyvinylpyrrolidone (Sigma Aldrich) as previously described [36]. For immunofluorescence staining, paws were frozen in OCT compound (Sakura Finetek USA, Torrance, CA, USA), cut into 8–12 mm sections in a cryostat (Leica CM1850, Wetzlar, Germany), and blocked with 5% BSA (Serva, Heidelberg, Germany) for 1 h at RT. To identify neutrophil infiltration, paws were stained with Rat anti-Ly6G antibody (BioLegend) or recommended Rat IgG2a Isotype control (BioLegend) overnight at 4 °C, followed by incubation with secondary Goat anti-Rat Alexa 647 antibody (Thermo Fisher) and DAPI (Sigma). Images were acquired using a Lionheart FX Microscope with 4× magnification (BioTek). Image stitching and deconvolution was performed using BioTek Gen5 Software for Imaging & Microscopy (Agilent). Quantification of the mean fluorescence intensity and analyzed area was performed with ImageJ (v1.54).

### 2.8. Cytokine Analyses from Synovial Fluid

7 days after K/BxN serum injection, mice were harvested as described above. SF was taken from the synovial cavity of both knees using Whatman papers as previously described [37]. In brief, skin was removed from the hind limb, the quadriceps muscle was cut at the proximal end, and the SF cavity was opened by retracting the quadriceps muscle with the patella downwards. Two Whatman papers were stacked in the cavity of each knee to allow resorption of SF and then stored in sterile 0.9% sodium chloride (NaCl, Braun) at 4 °C for 24 h. Afterwards, the whole fluid was extracted and stored at −80 °C. ELISAs of C5a and LTB4 (R&D Systems, Minneapolis, MN, USA) were performed according to the manufacturer’s protocols. Additionally, samples were sent to Olink (Thermo Fisher) for proteomic analysis (Olink Target 48 Mouse Cytokine panel). Olink relies on a proximity extension assay in combination with quantitative polymerase chain reaction readout and allows for reporting of both relative quantification (NPX value) as well as absolute quantification (pg/mL) based on calibrator curves [38]. Resulting absolute quantification was used for subsequent analysis, and log2 values were calculated after adding a small constant ϵ (1 × 10^−6^) to replace zero values in the dataset. RStudio (v2024.09.0+375) with the ggplot2 package was applied for principal component analysis (PCA), calculating 95% confidence ellipses of WT and WT control, and visualization. Heatmaps were generated as recently published [39]. Log2 values were standardized as z scores. The cut-off value for the z scores was defined as ±4 to prevent dominant effects of outliers on the color scale. The hierarchical clustering of samples and parameters and subsequent visualization were performed with RStudio, including the pheatmap package. Additionally, the absolute concentrations of selected cytokines were used to calculate percentage changes, using the mean value of each experimental group relative to WT.

### 2.9. Intravital Microscopy

Mice were anesthetized as stated above, and the cremaster muscle was prepared for intravital imaging as previously described [11,22,40,41,42]. Postcapillary venules with a diameter between 20 and 40 µm were investigated. To determine leukocyte adhesion, 500 ng CXCL1 (Peprotech, Cranbury, NJ, USA) were injected via the carotid artery. The number of adherent cells prior to and following CXCL1 injection was examined for 15 min. To determine selectin-mediated slow rolling, adhesion, and transmigration in vivo, mice were injected intrascrotally with 500 ng TNF (BioLegend) 2 h before the preparation of the cremaster muscle. Intravital microscopy was performed on an upright microscope (Axioskop, Zeiss) with a 40 × 0.75 NA saline immersion objective. Leukocyte rolling velocity and leukocyte adhesion were determined by transillumination intravital microscopy, whereas leukocyte extravasation was investigated by reflected light oblique transillumination microscopy as previously described [22,40,42]. Recorded images were analyzed using ImageJ and SlideBook version 5 (Intelligent Imaging Innovations). Emigrated cells were determined in an area 75 × 100 μm to each side of a vessel (representing 1.5 × 10^4^ µm^2^ tissue area). The microcirculation was recorded using a digital camera (Sensicam QE, pco.imaging, PCO AG, Kelheim, Germany).

### 2.10. Soluble ICAM-1- and Fibrinogen-Binding Assay

The soluble ICAM-1- and fibrinogen-binding assays were performed as previously described [43,44]. To assess LFA-1-specific ICAM-1 binding, isolated murine neutrophils were resuspended in HBSS containing 10 mM HEPES, 1 mM CaCl_2_, and MgCl_2_ and preincubated with a functional blocking anti-Mac-1 (clone M1/70, 10 μg/mL) antibody. Neutrophils were stimulated with CXCL1 (100 ng/mL, 3 min, 37 °C, Peprotech), LTB4 (150 ng/mL, 3 min, 37 °C, Cayman Chemical, Ann Arbor, MI, USA) or left untreated in the presence of anti-ICAM-1/Fc (20 μg/mL, clone B3.3) and APC-conjugated anti-human IgG1 (Southern Biotechnology, Homewood, AL, USA). Afterwards, neutrophils were fixed on ice (7.4% formaldehyde) and stained with Ly6B.2 antibody (Bio-Rad, Hercules, CA, USA). LFA-1-specific binding to ICAM-1/Fc was measured by flow cytometry (BD FACSCantoII, BD Biosciences, Franklin Lakes, NJ, USA). To investigate Mac-1 binding to fibrinogen, isolated murine neutrophils were incubated in 0.9% NaCl (0.1% glucose, 0.25% BSA, 2 mM HEPES) with 150 μg/mL Alexa 647-conjugated fibrinogen (Invitrogen, Waltham, MA, USA) and stimulated with CXCL1 (100 ng/mL, 10 min, 37 °C, Peprotech), LTB4 (150 ng/mL, 3 min, 37 °C, Cayman Chemical), or left unstimulated. Neutrophils were then stained with Ly6B.2 (Bio-Rad). Fluorescence intensity was measured by flow cytometry (BD FACSCantoII). Analysis of ICAM-1- and fibrinogen-binding assay were performed by FlowJo v10.

### 2.11. Surface Marker Expression upon Stimulation

Primary neutrophils were isolated from the BM via a 62% Percoll gradient (Sigma Aldrich) and resuspended in PBS (PAN) containing 1 mM CaCl_2_ and MgCl_2_. Cells were stimulated with CXCL1 (100 ng/mL, 10 min, 37 °C, Peprotech), LTB4 (150 ng/mL, 3 min, 37 °C, Cayman Chemical), or left untreated. After washing, cells were resuspended in Hanks’ buffer on ice and stained for spectral flow cytometry as described above.

### 2.12. Preparation of Plate-Bound Immune Complexes (ICs)

Preparation of ICs was performed as previously described [45]. Clear 96-well microplates (R&D Systems) were incubated with 200 µL human serum albumin (200 g/L, Behring), diluted 1:1000 in PBS for 45 min, followed by blocking with 10% FCS (PAN) in PBS for 1 h. After further incubation with anti-HSA antibody (∼10 mg/mL, Merck), diluted 1:1000 in PBS for 45 min, the plates were directly used for the indicated experiments. As a control, parallel wells were incubated with PBS in the absence of anti-HSA antibody.

### 2.13. ROS Production

Measurement of reactive oxygen species was performed as described previously [22,46]. Primary neutrophils were isolated from the BM and incubated in HBSS (Sigma Aldrich). Cells were plated on IC-coated or uncoated plates (Immulon 4HBX plates, Thermo Fisher) with CaCl_2_ (1 mM), MgCl_2_ (1 mM), and cytochrome c (0.1 mM, Sigma Aldrich) in the presence or absence of TNF (50 ng/mL, BioLegend) and/or superoxide dismutase (∼45 U, SOD, Sigma). Absorbance at 490 and 550 nm was recorded every 2 min for 90 min at 37 °C on a Synergy Mx plate reader (Bio-Rad). Data were analyzed by subtracting the SOD values from the corresponding values without SOD. Accumulated O_2_ production was summarized as the area under the curve (AUC), and mean values of each experimental group/genotype were used to calculate percentage changes relative to WT by dividing these values by that of WT.

### 2.14. LTB4 and IL-1ß Release in Vitro

Measurement of LTB4 and IL-1ß release after incubation on IC-coated plates was performed as previously described [5,47]. Primary neutrophils were isolated from the BM via a 62% Percoll gradient (Sigma Aldrich) and 1 × 10^6^ cells were incubated (37 °C, 5% CO_2_) in RPMI 1640 medium (Roswell Park Memorial Institute, w/o L-glutamine, w/o phenol red, w 2.0 g/L NaHCO_3_) with 2 mM L-glutamine, 1 mM CaCl_2_ and MgCl_2_ on IC-coated or control (Albumin) plates for 6 h. Afterwards, the cell suspension was centrifuged, and the supernatant was analyzed by LTB4 and IL-1ß ELISA (R&D Systems) according to the manufacturer’s protocol. Based on the mean values of each experimental group/genotype, percentage changes were calculated relative to the mean of WT. Viability was determined by staining the cell pellet with an anti-Ly6B.2 antibody (Bio-Rad) and DAPI (Sigma), followed by flow cytometry analysis (BD FACSCantoII).

### 2.15. Western Blotting

For biochemical assays, primary neutrophils were isolated and resuspended in PBS containing 10 mM each of CaCl_2_ and MgCl_2_. Cells were stimulated with CXCL1 (100 ng/mL, 2 min, 37 °C, Peprotech), LTB4 (150 ng/mL, 2 min, 37 °C, Cayman Chemical), TNF (50 ng/mL, 15 min, 37 °C, BioLegend), ICs (15 min, 37 °C, 5% CO_2_), or left untreated [5,11,22]. After stimulation, cells were directly lysed in radioimmunoprecipitation assay buffer for 10 min on ice, and lysates were boiled with sample buffer (10 min, 95 °C) as already described [22,48]. Cell lysates were run on 10% SDS-PAGE and immunoblotted using primary antibodies against indicated proteins (see resources table), followed by peroxidase-labeled secondary antibodies. Immunoblots were developed on an ECL system (enhanced chemiluminescence, Cytiva, Wilmington, DE, USA) using the ChemiDoc XRS+ system (Bio-Rad). Densitometric quantification was performed using Image Lab Software v6.1.0 (Bio-Rad).

### 2.16. Statistics

Data analysis and graphical representation were performed using Prism v9 and v10 software (GraphPad) unless otherwise described. All data are expressed as mean ± SEM, and n-numbers are detailed in the figure legends. Statistical significances were calculated (if indicated after ROUT outlier test, Q = 2%) as described in the figure legend using one-way or two-way ANOVA followed by Tukey post hoc test unless otherwise described. Data were considered statistically significant when *p* < 0.05.

## 3. Results

### 3.1. CD45 and CD148 Are Required for Onset and Progression of Serum Transfer-Induced Arthritis

**Figure 1:**
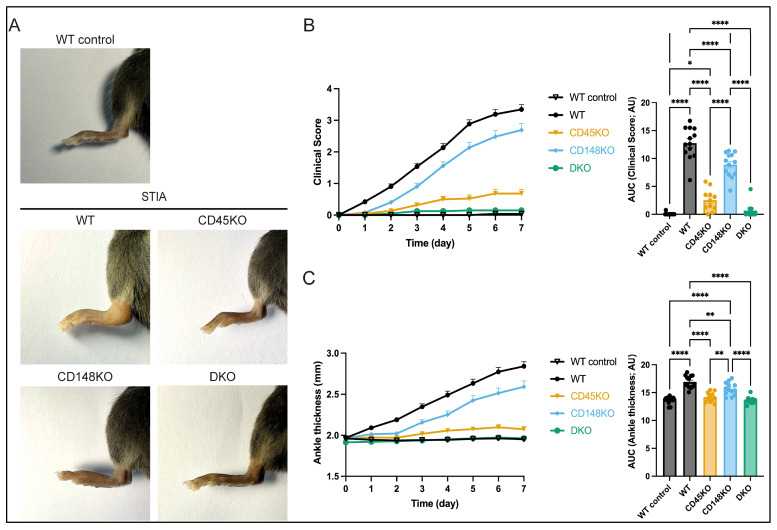
CD45 and CD148 are involved in onset and development of serum transfer-induced arthritis (STIA). WT, CD45KO, CD148KO, and DKO mice were injected with K/BxN serum and observed for 7 days. (**A**) Representative images of hind paws are shown. (**B**,**C**) Clinical score and ankle thickness were assessed daily (n = 10–13 mice in each group). All data are presented as mean +/− SEM, one-way ANOVA, * *p* < 0.05, ** *p* < 0.01, **** *p* < 0.0001.

We injected K/BxN serum into CD45KO, CD148KO, and DKO as well as C57BL/6J WT mice to analyze the role of CD45 and CD148 in a murine arthritis model. This model is neutrophil dependent, as has been previously demonstrated by the depletion of neutrophils, resulting in complete abrogation of arthritis [3,9,49]. As negative controls, we used WT mice that received an injection of 200 µL 0.9% NaCl (described as WT control). Clinical scores and ankle thickness were assessed over 7 days in our STIA model. CD45KO and, in particular, DKO mice did not develop arthritis when assessed by clinical scores and ankle thickness. In CD148KO mice, the onset of arthritis was delayed, and disease severity slightly decreased (Figure 1A–C).

**Figure 2:**
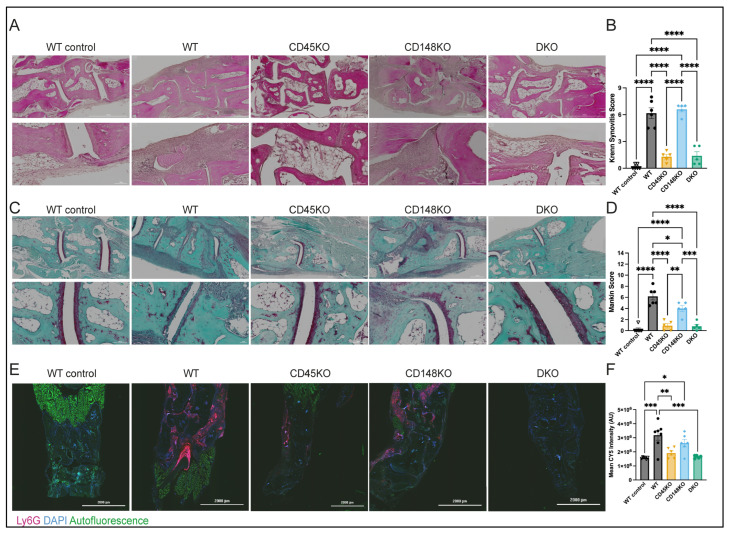
Synovitis and cartilage damage during STIA depend on CD45 and CD148. Mice were sacrificed and ankles harvested for histopathologic assessment 7 days after K/BxN serum injection. (**A**,**B**) Hematoxylin/Eosin (H/E) and Safranin O/Fast green staining were performed on paraffin-embedded sections. Representative images are shown with 4× (top) and 20× (bottom) magnification. (**C**,**D**) The degree of synovitis was assessed in H/E-stained sections using the Krenn Synovitis Score, cartilage damage in Safranin O/Fast green-stained sections according to Mankin Score (n = 5–6 mice in each group). (**E**,**F**) For analysis of recruitment of neutrophils into the ankles, immunofluorescence Ly6G staining of frozen tissue sections was performed, and mean fluorescence intensity was measured. Representative images are shown with 4x magnification (n = 5–7 mice per group). All data are presented as mean +/− SEM, one-way ANOVA, * *p* < 0.05, ** *p* < 0.01, *** *p* < 0.001, **** *p* < 0.0001.

Histological analyses of the hind paws from WT control, WT, CD45KO, CD148KO, and DKO mice were conducted using established scoring systems [34,35,50,51] and confirmed our clinical measurements (Figure 2A–D). Ankle joints of WT mice exhibited characteristic inflammatory infiltrates and cartilage damage. In accordance with the clinical data, almost no inflammatory infiltrates or cartilage damage were detected in CD45KO and DKO mice. Joints of CD148KO mice demonstrated inflammation comparable to WT mice, whereas cartilage damage was significantly reduced. To detect neutrophil recruitment to the ankles during STIA, we stained frozen tissue sections obtained at day 7 for Ly6G. Isotype control was used to verify specificity (Figure S1A). Mean fluorescence intensities were measured in representative ankle areas of similar size (Figure S1B,C). Herein, WT and CD148KO mice showed significant neutrophil infiltration, which was almost completely absent in CD45KO and DKO mice (Figure 2E,F). Additionally, we generated chimeric mice with CD45-, CD148-, or DKO-deficient bone marrow (BM) transplanted into WT animals to validate our previous results. Mice with CD45- and DKO-deficient BM showed almost no inflammation, whereas CD148-deficient BM reconstituted mice showed a moderate decrease compared to WT mice regarding clinical scoring and histological analyses (Figure S2A–C).

### 3.2. Presence of Proinflammatory Cytokines in Synovial Fluid During STIA Is Dependent on CD45

**Figure 3:**
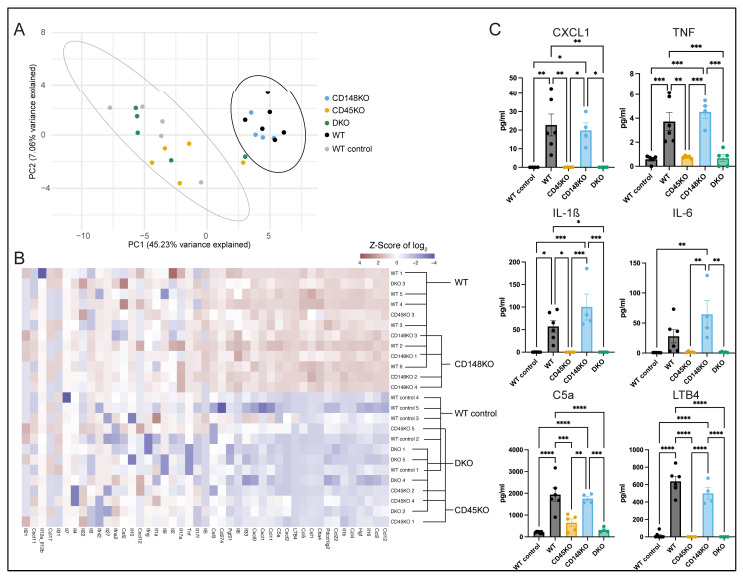
Proinflammatory chemokines/cytokines are strongly diminished in the SF of CD45KO and DKO mice. SF was collected from the knees 7 days after K/BxN serum injection. (**A**) Principal component analysis (PCA) with 95% confidence ellipses for WT control (grey) and WT (black) summarizes the differences in the chemokine/cytokine profile of each group. (**B**) A heatmap was generated to distinguish groups with similar or differing protein profiles and to identify proteins present in different amounts. Labeling of different genotype groups was added according to the 95% confidence ellipses (WT and WT control) of the PCA. (**C**) Selected chemokines/cytokines are shown with absolute quantification as individual graphs (ROUT = 2%, n = 4–6 mice in each group). All data are presented as mean +/− SEM, one-way ANOVA, * *p* < 0.05, ** *p* < 0.01, *** *p* < 0.001, **** *p* < 0.0001.

Because the recruitment of leukocytes into tissue compartments during inflammation is tightly regulated by a complex variety of cytokines, chemokines, and chemoattractants, we next set out to analyze the impact of CD45 and CD148 on the presence of these immunomodulatory cues in synovial fluid (SF) during inflammatory arthritis. Therefore, we isolated SF from both knees 7 days after K/BxN serum injection and performed targeted proteomic analyses for the detection of cytokines/chemokines paralleled by individual ELISA assays for the lipid mediator LTB4 and glycoprotein C5a, both acting as chemoattractants [52] (hereafter summarized as chemokine/cytokine profile). Based on principal component analysis (PCA), we noticed that the chemokine/cytokine profile of WT mice receiving K/BxN serum was clearly distinguishable from WT control mice receiving only NaCl. Interestingly, the chemokine/cytokine profile from the SF of CD148KO mice is similar to that of arthritic WT mice, whereas the profile of STIA-treated CD45KO and DKO mice appears to be similar to non-arthritic WT controls (Figure 3A). Focusing on specific chemokine/cytokine profiles, we found that chemokines/cytokines classically described as proinflammatory, such as CXCL1 and IL-6, are dominant in WT and CD148KO mice, clearly separating these from WT control, CD45KO, and DKO mice (Figure 3B). These results were confirmed by absolute quantification of selected proinflammatory chemokines/cytokines, which were differentially expressed between the groups (Figure 3C and Figure S3). For example, we found high levels of CXCL1 in SF of WT mice, which were relatively similar (calculated by using the mean value of each group relative to WT, WT: 100%) in CD148KO (87.02%), but significantly lower in WT control (0.16%), CD45KO (0.3%), and DKO (0.2%). We obtained comparable results for LTB4 (WT control: 2.61%, WT: 100%, CD45KO: 0.0%, CD148KO: 78.36%, DKO: 0.0%) and C5a (WT control: 10.71%, WT: 100%, CD45KO: 33.62%, CD148KO: 90.51%, DKO: 16.0%).

### 3.3. Synovial Fluid of WT and CD148KO Mice Contains High Amounts of Neutrophils with an Activated Proinflammatory Phenotype

**Figure 4:**
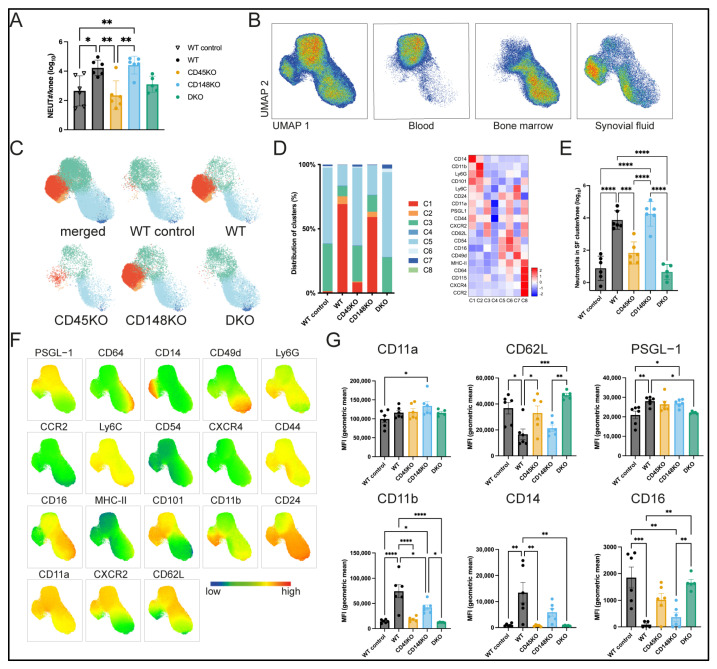
Neutrophils in SF from WT and CD148KO mice show an activated proinflammatory phenotype, which is absent in CD45KO and DKO mice. Neutrophils were isolated 7 days after K/BxN serum injection and analyzed by spectral flow cytometry. (**A**) Neutrophil counts in SF from both knees were determined for each genotype. (**B**) UMAP analysis was performed upon the neutrophil gate for the visualization of neutrophils from blood, BM, and SF. (**C**) Based on UMAP analysis, clustering of neutrophils was performed and visualized here only for SF samples according to each genotype. (**D**) Cluster proportion and heatmap attribution of neutrophils only from SF according to genotype. (**E**) Neutrophil counts in the identified activated, proinflammatory clusters are shown. (**F**) Expression level of surface markers projected onto UMAP plot. (**G**) Selected surface markers are presented separately (ROUT = 1%, n = 5–6 mice in each group). All data are presented as mean +/− SEM, one-way ANOVA, * *p* < 0.05, ** *p* < 0.01, *** *p* < 0.001, **** *p* < 0.0001.

Next, we focused on the surface phenotype of neutrophils recruited into SF during inflammatory arthritis. Seven days after K/BxN serum injection, neutrophils (CD11b+Ly6CintCD115-Ly6G+ cells; applied gating strategy in Figure S4A) from SF, blood, and BM were analyzed by spectral flow cytometry. First, we determined absolute neutrophil counts in the SF from both knees for each genotype and identified a marked recruitment of neutrophils in WT and CD148KO mice, which were absent in mice without K/BxN serum injection, in line with our histological analyses (Figure 4A and Figure S4B,C). Using Uniform Manifold Approximation and Projection (UMAP) analysis employing all markers described in Figure 4F, we were able to show that neutrophils displayed a distinct clustering between tissues (Figure 4B). Focusing on synovial fluid neutrophils, unsupervised clustering showed that neutrophils in the SF comprise activated clusters with regard to the differentially regulated markers (Clusters C1–C2, Figure 4C–F). These activated clusters were present in the SF of WT and CD148KO mice, whereas SF neutrophils in CD45KO and DKO mice resembled a phenotype similar to blood and BM neutrophils, showing reduced activation (Clusters C3–C8, Figure 4D,E and Figure S4D–F). Regarding the differentially expressed surface markers of neutrophils from SF samples (Figure 4G and Figure S4G), we additionally observed that CD45KO and DKO neutrophils exhibited reduced expression of CD11b and CD14, whereas these strains showed persistently high CD62L surface levels, comparable to WT control animals. In CD148KO mice, an intermediary activation phenotype was found with regard to CD11b and CD14 surface levels.

### 3.4. CD45 Deficiency Results in Impaired Neutrophil Recruitment, Differential β2-Integrin Activation, and Affectation of Effector Functions

**Figure 5:**
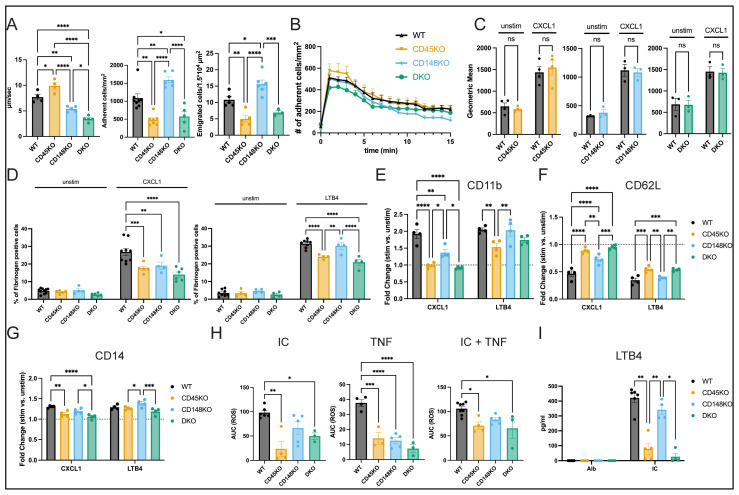
CD45 shows overarching modulation of neutrophil recruitment and effector functions compared to CD148. (**A**) Mice were injected intrascrotally with TNF, and rolling velocity, number of adherent and emigrated cells were assessed after 2 h (n = 3–8 mice in each group). (**B**) For analysis of chemokine-induced arrest, the cremaster muscle was prepared, and CXCL1 was administered via a carotid artery catheter (n = 3–5 mice in each group). Binding of the β2-integrin ligands ICAM-1 (**C**) and fibrinogen (**D**) was assessed by flow cytometry after BM-derived neutrophils were stimulated with CXCL1 or LTB4 (n = 3–10 mice in each group). (**E**–**G**) BM-derived neutrophils were stimulated with CXCL1 or LTB4 and analyzed for expression of indicated surface proteins (n = 4 mice in each group). (**H**) BM-derived neutrophils were plated on uncoated or IC-coated plates with or without TNF, and ROS production was measured. Accumulated O_2_ production was summarized as area under the curve (n = 3–8 mice in each group). (**I**) LTB4 was measured in the supernatants by ELISA after BM-derived neutrophils were incubated on IC-coated plates for 6 h. Albumin (Alb)-coated plates were used as controls (n = 3–6 mice in each group). All data are presented as mean +/− SEM, one and two-way ANOVA, ns = not significant, * *p* < 0.05, ** *p* < 0.01, *** *p* < 0.001, **** *p* < 0.0001.

To further study the involvement of CD45 and CD148 in the different steps of the recruitment cascade, we performed intravital microscopy of the TNF-inflamed cremaster muscle (Figure 5A and Figure S5A). Herein, a significant increase in neutrophil rolling velocities in CD45KO mice was visible, whereas CD148KO and DKO mice showed a decreased velocity. The number of adherent and transmigrated neutrophils was significantly lower in CD45KO and increased in CD148KO animals, suggesting that CD45 and CD148 play complementary roles in neutrophil recruitment in vivo. To investigate whether CD45 and CD148 are involved in chemokine-induced arrest, we imaged the cremaster muscle before and after injection of CXCL1, which typically leads to immediate neutrophil arrest mediated by lymphocyte function-associated antigen-1 (LFA-1) [22]. WT, CD45KO, CD148KO, and DKO mice showed no difference in the number of adherent neutrophils with unaltered neutrophil counts in untreated mice (Figure 5B and Figure S4C). To confirm these results in vitro, we examined intercellular adhesion molecule-1 (ICAM-1) binding as a readout of LFA-1 activation. Unstimulated as well as CXCL1-stimulated neutrophils of each genotype bound equal amounts of ICAM-1 (Figure 5C). As a readout for Mac-1 (macrophage antigen-1) in vitro activity, we measured the percentage of fibrinogen-positive unstimulated and CXCL1-stimulated neutrophils. CD45KO, CD148KO, and DKO neutrophils bound significantly less fibrinogen compared to WT neutrophils upon CXCL1 stimulation (Figure 5D). LTB4 has been shown to control neutrophil infiltration into joints and the development of STIA [47,53,54]. Concordantly, we detected high concentrations of LTB4 in the SF of WT and CD148KO mice (Figure 3C). Therefore, we performed the same in vitro assays, stimulating neutrophils with LTB4. Following stimulation with LTB4, PMNs of each genotype bound equal amounts of ICAM-1 (Figure S5B), but the amount of bound fibrinogen differed between genotypes (Figure 5D). CD45KO and DKO neutrophils bound significantly less fibrinogen, whereas CD148KO neutrophils were able to bind similar amounts of fibrinogen compared to WT. To investigate stimulation-dependent surface marker expression and assess whether we could mimic phenotypic changes of SF neutrophils (Figure 4) in vitro, we stimulated neutrophils isolated from BM with CXCL1 and LTB4 and analyzed the surface expression of selected markers by spectral flow cytometry. In line with the fibrinogen-binding response, a stimulus-dependent regulation of surface CD11b as well as CD62L levels was observed (Figure 5E,F). Following CXCL1 stimulation, a significantly impaired regulation of surface expression of CD11b and CD62L in CD148KO, as well as CD45KO and DKO neutrophils, could be observed compared to WT stimulated cells. In contrast, following LTB4 stimulation, the regulation of expression was only impaired in CD45KO and DKO neutrophils (Figure 5E,F). CD14 expression was significantly lower in CD45KO and DKO neutrophils after CXCL1 stimulation, in concordance with observations from SF neutrophils (Figure 4G and Figure 5G). After being recruited into inflamed joints, neutrophils contribute to joint damage and propagation of immune responses through the production of ROS and release of cytokines. ROS production and cytokine release were determined in the presence of immune complexes (ICs) [5,47]. In the presence of ICs and/or TNF, ROS production was reduced in CD45KO (IC: 23.85%, TNF: 37.17%, IC + TNF: 67.12%), CD148KO (IC: 67.52%, TNF: 33.12%, IC + TNF: 78.9%) and DKO (IC: 50.59%, TNF: 19.07%, IC + TNF: 61.86%) neutrophils compared to WT (IC, TNF and IC+TNF each: 100%) (Figure 5H and Figure S5C). It has previously been shown that neutrophils stimulated with ICs are capable of releasing cytokines, including LTB4 and IL-1β. Consequently, we investigated whether the release of these cytokines was impaired in the absence of CD45 and/or CD148. Indeed, LTB4 release was significantly reduced in CD45KO (18.49%, mean value relative to WT) and DKO (6.26%) neutrophils, whereas CD148KO neutrophils showed no significant reduction (81.16%) (Figure 5I). Contrasting, IL-1β release was rather increased in CD45KO and DKO neutrophils but decreased in CD148KO neutrophils, although not reaching significance (Figure S5D). Comparable high levels of cellular viability across all genotypes were confirmed using flow cytometry, corroborating that this observation was not due to cell death artefacts (Figure S5E).

### 3.5. CD45 and CD148 Have Distinct Regulatory Effects on GPCR- and Fc-Mediated Signaling

**Figure 6:**
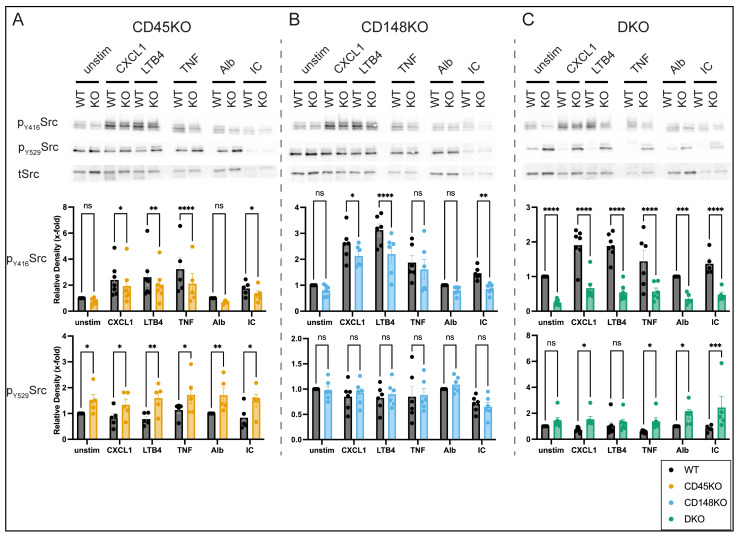
SFK phosphorylation is differentially affected by CD45 and CD148. BM-derived neutrophils were left untreated or stimulated with CXCL1 or LTB4 for 2 min or TNF for 15 min. Additionally, BM-derived neutrophils were incubated on IC-coated plates for 15 min. Alb-coated plates were used as controls. Cells were lysed and immunoblotted with Ab against total-Src, phospho-Src Y416, or Y529. Quantification of the phosphorylated proteins as a relative density of total protein, normalized to untreated or Alb-coated lysates, was performed. Representative Western blots of total lysates and quantification from CD45KO (**A**), CD148KO (**B**), and DKO (**C**) neutrophils, each compared to WT, showing the phosphorylation of SFKs (n = 5–7 mice in each group). All data are presented as mean +/− SEM, two-way ANOVA followed by Fisher’s LSD test, ns = not significant, * *p* < 0.05, ** *p* < 0.01, *** *p* < 0.001, **** *p* < 0.0001.

**Figure 7:**
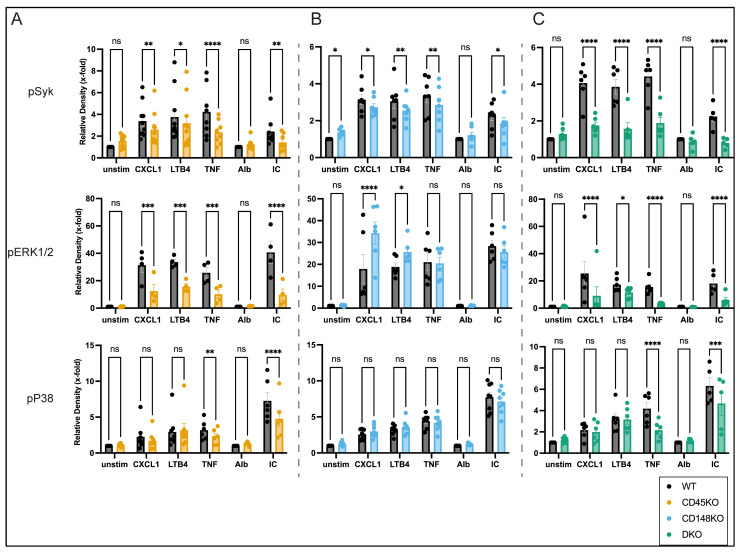
CD45 and CD148 are differentially involved in GPCR- and Fc-mediated signaling. BM-derived neutrophils were left untreated or stimulated with CXCL1 or LTB4 for 2 min or TNF for 15 min. Additionally, BM-derived neutrophils were incubated on IC-coated plates for 15 min. Therefore, Alb-coated plates were used as controls. Cells were lysed and immunoblotted with Ab against total-Syk, phospho-Syk, total-P38, phospho-P38, total-ERK1/2, and phospho-ERK1/2. Quantification of the phosphorylated proteins as a relative density of total protein, normalized to untreated or Alb-coated lysates, is shown for CD45KO (**A**), CD148KO (**B**), and DKO (**C**) neutrophils, each compared to WT (n = 4–10 mice in each group). All data are presented as mean +/− SEM, two-way ANOVA followed by Fisher’s LSD test, ns = not significant, * *p* < 0.05, ** *p* < 0.01, *** *p* < 0.001, **** *p* < 0.0001.

Next, we investigated the distinct disease-related cytokine-, GPCR-, and Fc-mediated signaling mechanisms of CD45 and CD148 involved in integrin activation as well as regulation of effector functions. For this purpose, stimulation of isolated neutrophils was performed using CXCL1, LTB4, and TNF, whereas ICs were utilized to engage Fc-mediated signaling [47,55]. Subsequently, the activation status of SFKs, Syk, ERK1/2 (also known as P44/42 MAPK), and P38 was determined (Figure 6, Figure 7 and Figure S6). It has been previously shown that SFKs are critically involved in the development of STIA and that both CD45 and CD148 regulate SFK activity, yet details with regard to overlapping or diverging signaling pathways still remain unclear [5,22,24]. In CD45KO neutrophils, GPCR- and Fc-mediated activation as well as TNF stimulation led to decreased phosphorylation of SFKs at Y416, whereas phosphorylation of the inhibitory Y529 was increased compared to WT (Figure 6A). CD148KO neutrophils also showed reduced phosphorylation of SFKs at Y416, but in contrast to CD45KO, phosphorylation of SFKs at Y529 was not altered (Figure 6B). Stimulation of DKO neutrophils resulted in a more marked reduction in phosphorylation of SFKs at Y416 and an increased phosphorylation of SFKs at Y529 (Figure 6C).

We further investigated the activation status of Syk, ERK1/2, and P38 as potentially involved signaling pathways that have critical functions in the activation of neutrophils. Importantly, Syk and P38 (reduced development of STIA in Syk-deficient or P38 inhibitor-treated mice), as well as ERK1/2 (Fc-receptor stimulation leads to ERK1/2 activation), have been implicated in the context of arthritis [56,57,58,59]. In CD45KO neutrophils, phosphorylation of Syk and ERK1/2 was reduced for all tested stimuli, while activation of P38 was only decreased after incubation with TNF and ICs (Figure 7A and Figure S6A). In CD148KO, Syk activation was also diminished compared to WT neutrophils, whereas phosphorylation of ERK1/2 was increased after GPCR-mediated activation. Furthermore, CD148KO neutrophils showed no reduced phosphorylation of P38 (Figure 7B and Figure S6B). Similar to CD45KO and CD148KO cells, stimulation of DKO neutrophils by GPCR, TNF, and Fc-receptor led to reduced Syk phosphorylation (Figure 7C and Figure S6C). DKO neutrophils also showed decreased ERK1/2 activation and stimulus-dependent decreased phosphorylation of P38, comparable to CD45KO neutrophils.

## 4. Discussion

The involvement of the RPTPs CD45 and CD148 in neutrophil recruitment and function in the context of autoimmune diseases is insufficiently characterized. Using a murine model of serum transfer-induced arthritis, we identified CD45 as a critical regulator of neutrophil recruitment and effector functions. For CD148, we found a more complex role in the onset and progression of arthritis. Herein, the onset of STIA was delayed, but neutrophil recruitment into the joints was not abrogated, while ROS production as a typical effector function was significantly diminished. Thus, CD45 and CD148 share functions as positive regulators of neutrophil functionality, whereas CD148 appears to feature additional unique purposes as a negative regulator.

**Figure 8:**
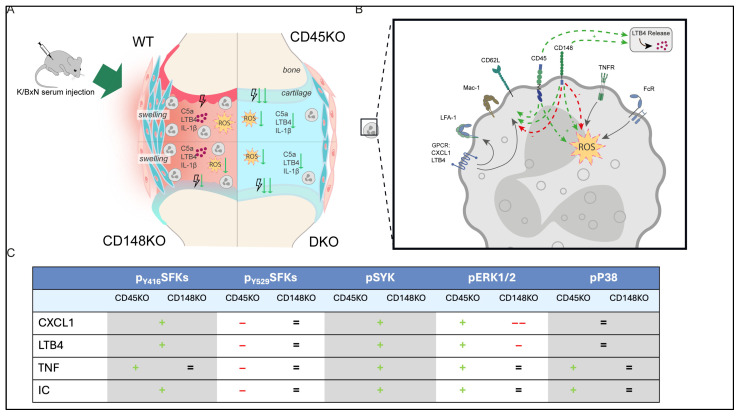
Schematic model of the role of CD45 and CD148 during inflammatory arthritis. (**A**) After injection of K/BxN serum into CD45KO, CD148KO, DKO, as well as C57BL/6J WT mice, CD45 and CD148 feature distinct regulative properties regarding clinical and histological parameters, presence of cytokines, as well as neutrophil recruitment and functionality. Based on these properties, CD45KO and, in particular, DKO lead to an overall reduction in the development of inflammatory arthritis, whereas CD148KO results in reduced ROS production and cartilage damage but unaltered cytokine release and overall neutrophil infiltration. (**B**) On a cellular level, upon GPCR-mediated stimulation, Mac-1 activation and selectin engagement are positively (+) mediated by CD45 and both positively and negatively (−) mediated by CD148, whereas LFA-1 activation is independent of CD45 or CD148. Additionally, CD45 and CD148 are required for ROS production and LTB4 release after TNF- and Fc-mediated stimulation. (**C**) At a molecular level, signaling pathways are regulated in distinct ways. Activation of SFKs (detected by pY416) and Syk is positively regulated by both CD45 and CD148, while dephosphorylation of inactivated SFKs (pY529) is only dependent on CD45. The activation of ERK1/2 is positively regulated by CD45 and negatively by CD148, with particularly strong regulation after stimulation with CXCL1. P38 is only positively regulated by CD45 in a stimulus-dependent manner after TNF- and Fc-mediated activation.

Following K/BxN serum transfer, ICs in the joints induce inflammation by complement activation and cytokine production [7,60]. Thereby, neutrophils are the key effector cells recruited into the joints and the main producers of proinflammatory cytokines, including LTB4 and CXCL1/2 [3,7,49,61]. Although these processes are well studied, little is known about the positive and negative regulation during inflammatory arthritis. Our results show that CD45 positively regulates neutrophil recruitment into the joint and is positively involved in the initiation of the lipid–cytokine–chemokine cascade, as LTB4, IL-1ß, and downstream chemokines were significantly decreased in CD45KO [47,55]. In contrast, CD148KO results in delayed but not abolished neutrophil recruitment and an overall proinflammatory chemokine/cytokine pattern comparable to WT, indicating a minor role for CD148 in generating an inflammatory microenvironment. Together with our data on LTB4 release in vitro, we provide further evidence that Fc-receptor-mediated generation of cytokines/lipids serves as an important factor in the K/BxN STIA model, enhancing neutrophil recruitment into inflamed tissue [4,5,47]. Regarding the different phases of neutrophil recruitment, consistent with the study by Germena et al. [22], we demonstrate that selectin-dependent as well as Mac-1-activation-dependent recruitment steps are diminished in the absence of CD45, contributing to an overall reduced neutrophil recruitment. However, LFA-1 activation is not altered in CD45KO neutrophils. It is possible that the chemokine-induced arrest is not sensitive enough to detect these differences. Interestingly, contrasting observations were made in CD148KO mice, indicating an enhanced activation in line with previously described results in the *S. aureus* model [24]. Consistent with these studies implicating CD45 and CD148 in integrin activation and involvement in infection models [22,24], our data suggest that CD148 has stimulus-dependent negative and positive effects on neutrophil recruitment regarding selectin engagement and Mac-1 activation, whereas CD45 has mainly positive effects (graphically presented in Figure 8).

Emphasizing disease- and tissue-specific mechanisms of neutrophil recruitment, these implications may have different importance in autoimmune-induced joint inflammation [5,8,62,63]. In our study, the K/BxN STIA model was chosen due to its rapid onset and neutrophil-dependence. Future studies are warranted to assess cross-model regulative aspects of CD45 and CD148, also utilizing other more chronic models, such as collagen-induced arthritis. Accordingly, in the K/BxN STIA model, LFA-1 was shown to be critical for neutrophil recruitment, while Mac-1 is not. In contrast, in the K/BxN TCR transgenic mouse and a rat adjuvant arthritis model, knockout/blockade of LFA-1 or Mac-1 was ineffective [64,65,66]. Additionally, in a model of reverse passive Arthus reaction, leukocyte infiltration was reduced in L-selectin-deficient mice [67]. Therefore, we hypothesize that CD45 is involved in regulating neutrophil recruitment to inflamed tissue through selectin engagement and Mac-1 activation, as well as generation of an inflammatory microenvironment, with the effectual combination contributing to a defective recruitment in CD45KO [5,42,68]. In contrast, CD148 is stimulus-dependent and also involved in selectin engagement and Mac-1 activation with less pronounced effects on cytokine production/release, resulting in a delayed but overall unaltered neutrophil infiltration in CD148KO. This emphasizes the importance of the generation of proinflammatory chemokines/cytokines in the STIA model but also suggests that the initiation of recruitment is impaired in CD148KO, whereas amplification by redundant/alternative mechanisms remains unaltered [4,5,64]. However, further experiments, such as the adoptive transfer of WT and KO neutrophils, may aid in examining overlapping functions of dysregulated cytokine expression and decreased neutrophil recruitment. Determining the chemokine/cytokine profile and neutrophil recruitment at earlier time points of arthritis will additionally help to identify potential differences in the kinetics of CD148KO mice in future studies.

To further reveal the redundant and non-redundant functions of CD45 and CD148 on a molecular level, we focused on key neutrophil signaling pathways (schematically summarized in Figure 8). It has been described that these RPTPs are involved in the activation of SFKs [22,23,24,27]. Previous studies showed that mice lacking the myeloid SFKs or the Syk tyrosine kinase, which are involved in pathways downstream of Fc receptors and SFKs, are completely protected against the development of arthritis in the K/BxN STIA model [5,56,69]. Nevertheless, the exact mechanisms are not understood. SFKs and Syk have been attributed key regulatory roles in migration, β2 integrin–mediated neutrophil activation and recruitment, as well as ROS production [5,70,71,72]. Previous work on CD45 and CD148 showed, for instance, an increased Y416 SFK phosphorylation after E-selectin engagement in neutrophils carrying a single point mutation (CD45E613R) that constitutively activates CD45 [22]. In line with this, CD45KO neutrophils showed reduced activation of all SFKs, as well as reduced AKT and ERK1/2 activation after fMLF stimulation, whereas CD148 showed preferential recognition of Lyn, which was reflected by increased AKT and ERK1/2 activation [24]. For the MAPKs ERK1/2 and P38, it has been demonstrated that these signaling proteins are involved in β2-integrin- and Fc-receptor-mediated processes, neutrophil recruitment, chemotaxis, and cytokine production [57,73,74,75,76]. Combining our data on GPCR-mediated signaling with our functional results regarding neutrophil recruitment, we suggest that Mac-1 activation following GPCR-mediated stimulation and L-selectin shedding are strictly dependent on CD45, CD148, and downstream activation of SFKs and Syk, whereas LFA-1 activity is independent. Nonetheless, the role of CD148 appears to be more complex, as a stimulus-dependent regulation of Mac-1 expression and L-selectin shedding was observable. Regarding neutrophil effector functions such as phagocytosis and ROS production, it was previously shown that these are inhibited in CD45KO and CD148KO neutrophils [22,24]. Interestingly, we found that TNF and Fc-mediated stimulation led to an impaired activation of SFKs at Y416 and Syk in CD45KO and CD148KO neutrophils, which was even more pronounced in DKO neutrophils. These data correlate well with our ROS assay and indicate that CD45 and CD148, through activation of SFKs and Syk, are critically involved in neutrophil ROS production upon TNF and Fc-receptor stimulation [24,45,72,77]. Focusing on cytokine production/release, previous studies demonstrated that cytokine production in neutrophils is dependent on SFKs and P38 [5,75,78]. In the K/BxN STIA model, P38 inhibition resulted in reduced development of arthritis and expression of proinflammatory cytokines [58]. In line with that, a decreased MAPK activation and increased inhibitory phosphorylation of SFKs (Y529) following Fc-mediated stimulation were only present in CD45KO and DKO neutrophils, which also showed significantly reduced LTB4 release in vitro. These data suggest that CD45 is critically involved in cytokine release via MAPK and SFKs. In contrast, it can be summed up that CD148 shows overall stimulus dependence across different stimuli tested. As chemokinetic signalling-responses via GPCRs are indeed similarly modulated by CD148, these effects are opposingly or non-concomitantly regulated for other receptor classes, including responses to TNF or Fc-receptor stimulation. The mechanisms for this response are currently unclear but apparently evoke downstream of receptor-interaction by co-receptor signalling. Additional inhibitor experiments specifically targeting the analyzed signaling pathways will be needed with further investigation of the specific effects on neutrophil functions such as recruitment, ROS production, and cytokine release. These experiments would further help to emphasize the dual role of CD148 as a negative/positive regulator. Functionally, though, for example, ROS production as well as Mac-1 functionality are dually negatively and positively regulated by CD148, depending on the stimulus. At a molecular level, activation of SFKs and Syk is positively regulated by CD148, whereas ERK1/2 activation is negatively regulated, and dephosphorylation of inactivated SFKs and P38 activation are independent of CD148. These findings demonstrate the complexity and necessity of future studies to dissect the precise interplay of stimuli, downstream pathway intersections, and resulting functional impairments or enhancements in neutrophils.

Although neutrophils are crucial contributors to inflammatory arthritis, arthritis pathogenesis is complex and also involves other immune cells and a multitude of inflammatory mediators. Importantly, CD45 and CD148 are not only expressed in neutrophils but also in other leukocyte and lymphocyte subsets. In this current study, bone marrow chimeric mice have been created together with substantial in vitro work in order to clarify the role of CD45 and CD148 in neutrophils. Bone marrow chimeric mice can be helpful in differentiating recruited vs. local resident cell profiles as well as endo-/epithelial or fibroblast contributions. Nonetheless, the observed phenotypes may also be impacted by BM-transplantation-derived macrophages or mast cells. Thus, future studies are warranted to confirm such observations and dissect in detail the specific in vivo function of CD45 and CD148 in all leukocyte and lymphocyte populations using adoptive transfer methodology and conditional KO strategies. Different mediators have been described in the disease initiation and progression of inflammatory arthritis, including C5a and LTB4. Our experiments show that modulation of C5a and LTB4 levels is detectable during STIA depending on the presence/absence of CD45 and CD148. Similarly, distinct mechanistic responses following LTB4 stimulation can be observed (compare Figure 5 and Figure 6). Nonetheless, further studies are required to assess the mechanistic interplay in further detail, especially with regard to complement pathway activation and modulation of LTB4 responses.

Targeted drug therapy has emerged as a major advancement in recent years, offering a more precise and individualized approach to disease treatment. Our work thereby provides a so far unrecognized perspective for cell-specific disease-relevant effects of targeting CD45 and/or CD148. In this context, a limited number of studies have focused on the role of RPTPs CD45 and CD148 in neutrophil recruitment or function. However, these studies mainly used infection models and not models of autoimmune disease [22,24].

Indeed, during *E. coli* lung infection, the number of neutrophils in the alveolar compartment was significantly reduced but increased in the interstitial compartment, suggesting that CD45E613R neutrophils have a defect either in migratory capacity or in the ability to transmigrate across the epithelial barrier [22]. These results are consistent with another study in which the absence of CD45 or CD148 led to impaired integrin-mediated cell adhesion [24]. These data suggest that blocking CD45 may not be beneficial for treating infectious diseases, because pathogens cannot be fought effectively, which could lead to worse outcomes in such a setting.

In contrast, in settings of autoimmunity, the fact that the absence or, in a therapeutic context, the blocking of the CD45 or CD148 axes modulates and partially hinders neutrophils from recruiting into tissues is presumably advantageous. Of importance, the differing and stimulus-dependent interplay of CD45 and CD148 offers the unique opportunity to target specific components of recruitment or activation steps. In this context, reduced neutrophil recruitment and effector functions could prevent an excessive immune reaction. Indeed, phosphatase inhibitors and isoform-specific antibodies against CD45 have been developed, but these have only been tested in the context of leukemia, organ transplantation, or IgE-mediated anaphylaxis [79,80,81,82]. Their use in autoimmune diseases is still pending, but given the results of our study, this could be one essential step to address. Pathomechanistically, rheumatoid arthritis is associated with inflammation, tissue damage, and cartilage destruction [83]. Likewise, ROS levels are elevated in diseases such as rheumatoid arthritis and have been linked to tissue remodeling [84]. Notably, high ROS levels can trigger cell death pathways, resulting in cellular apoptosis and necroptosis and thus tissue destruction, whereas low ROS levels rather exhibit tissue- and immune-modulatory features [85]. Therefore, additional studies are warranted to assess the interplay of CD45/CD148 impacted ROS-responses during arthritis and its implications for localized cell death. Furthermore, rheumatoid arthritis pathogenesis is complex and depends on a variety of different immune cell populations. Implications of Th1/Th17 cells have been demonstrated [86]. Therefore, future studies are warranted to also assess the relevance of CD45/CD148 effects during the course of disease for these cellular subsets. Most importantly, therapeutic modalities are needed to approach this complex disease, also via distinct targeted delivery approaches [87].

## 5. Conclusions

In conclusion, our study demonstrates an important role for CD45 and CD148 in the context of neutrophil functionality in a murine model of inflammatory arthritis. As a positive regulator, CD45 is critically involved in neutrophil recruitment to inflamed tissue as well as in ROS production and cytokine release through activation of SFKs and Syk. In contrast, CD148 serves as a dual positive-negative regulator with redundant function in ROS production and unaltered cytokine release, whereas neutrophil recruitment appears to be stimulus-dependently diminished by activation of SFKs and Syk. These findings of a clear positive regulator and a stimulus-dependent dual positive-negative regulator may help in the future to fine-tune locally needed specific responses, since neutrophil activation is crucial for primary immune defence but detrimental during autoimmunity. Thus, delicate balancing of these immune responses is crucially needed and might be a target for future cell-specific therapeutic interventions, including autoimmune diseases such as rheumatoid arthritis.

## Data Availability

The original contributions presented in this study are included in the article/Supplementary Materials. Further reasonable inquiries can be directed to the corresponding author.

## Supplementary Materials

The following supporting information can be downloaded at https://www.mdpi.com/article/10.3390/cells14151169/s1, Table S1: resources table; Figure S1: immunofluorescence Ly6G staining of ankle frozen tissue sections; Figure S2: hematopoietic deficiency of CD45 and CD148 partially prevents from onset and progression of serum transfer induced arthritis; Figure S3: concentration of IL-10 and CXCL2 in SF of WT, CD45KO, CD148KO and DKO mice; Figure S4: gating strategy for neutrophils from BM, blood and SF and neutrophil counts of STIA and untreated WT, CD45KO, CD148KO and DKO mice; Figure S5: ICAM-binding assay, ROS production, and determination of viability during incubation with ICs; Figure S6: representative Western blots of total lysates after GPCR- and Fc-mediated stimulation showing phosphorylation of Syk, ERK1/2 and P38.

## Abbreviations

The following abbreviations are used in this manuscript:

CD	cluster of differentiation
CXCL	C-X-C motif chemokine ligand
LFA-1	lymphocyte function-associated antigen-1
Mac-1	Macrophage-1 antigen
ICAM	intercellular adhesion molecule
LTB4	Leukotriene B4
KO	knockout
DKO	double knockout
STIA	serum transfer-induced arthritis
PMN	polymorphonuclear neutrophils
SF	synovial fluid
BM	bone marrow
IC	immune complexes
GPCR	G-protein coupled receptor
SFK	Src family kinases
TNF	tumor necrosis factor
RPTPs	receptor-like tyrosine phosphatases
