# Supplementary material for: CD45 and CD148 Are Critically Involved in Neutrophil Recruitment and Function During Inflammatory Arthritis in Mice
Source: Cells. 2025 Jul 29;14(15):1169. doi: 10.3390/cells14151169 (PMC12346378; doi:10.3390/cells14151169)
Supplement: Supplementary file 1 [file cells-14-01169-s001.zip › cells-3760254-supplementary.pdf]

Supplementary Material

Supplementary Tables

Supplemental Table S1: resources table

REAGENT or RESOURCE	SOURCE	IDENTIFIER
**Antibodies**		
anti-mouse-Ly6G-BUV395	BD Biosciences	Cat#565964; RRID: AB_2716852
anti-mouse-CD11b-BUV496	BD Biosciences	Cat#749864; RRID: AB_2874105
anti-mouse-CD49d-BUV563	BD Biosciences	Cat#741243; RRID: AB_2870794
anti-mouse-I-A/I-E/MHCII-BUV661	BD Biosciences	Cat#750280; RRID: AB_2874471
anti-mouse-CD115-BUV737	BD Biosciences	Cat# 750948; RRID: AB_2875029
anti-mouse-CD162/PSGL-1-BV421	BD Biosciences	Cat#562807; RRID: AB_2737808
anti-mouse-CD62L-BV480	BD Biosciences	Cat#746726; RRID: AB_2743990
anti-mouse-CD44-BV570	BioLegend	Cat#103037; RRID: AB_10900641
anti-mouse-CXCR4-BV711	BioLegend	Cat#146517; RRID: AB_2687244
anti-mouse-CCR2-BV750	BD Biosciences	Cat# 747967; RRID: AB_2872428
anti-mouse-Ly6C-BV785	BioLegend	Cat#128041; RRID: AB_2565852
anti-mouse-CD11a-FITC	BioLegend	Cat#101106; RRID: AB_312779
anti-mouse-CXCR2-PerCP-Cy5.5	BioLegend	Cat# 149606; RRID: AB_2565565
anti-mouse-CD54-PE	BioLegend	Cat#116108; RRID: AB_313699
anti-mouse-CD16-PE-Dazzle 594	BioLegend	Cat#158012; RRID: AB_2876543
anti-mouse-CD64-PE-Cy7	BioLegend	Cat#139314; RRID: AB_2563904
anti-mouse-CD101-APC	Thermo Fisher	Cat#17-1011-82; RRID: AB_2815082
anti-mouse-CD24-Alexa 700	BioLegend	Cat#101836; RRID: AB_2566730
anti-mouse-CD14-APC-Fire 750	BioLegend	Cat#123332; RRID: AB_2734180

anti-mouse-Ly-6G	BioLegend	Cat#127602; RRID: AB_1089180
rat IgG2a, κ Isotype Ctrl Antibody	BioLegend	Cat#400502; RRID: AB_326523
anti-Rat-IgG Secondary Antibody, Alexa 647	Thermo Fisher	Cat#A48265; RRID: AB_2895299
anti-Mac-1 (clone M1/70)	purified from hybridoma supernatant	N/A
anti-ICAM-1/Fc (clone B3.3)	purified from hybridoma supernatant	N/A
anti-human-IgG1-APC	Southern Biotechnology	Cat# 9042-11; RRID: AB_2796610
anti-mouse-Ly-6B.2 (7/4)-FITC	Bio-Rad	Cat#MCA771FB; RRID: AB_322951
anti-HSA (human serum albumin)	Merck	Cat#MABX1933-1KC
phospho-Src Family Tyr416 (D49G4)	Cell signaling	Cat#6943S
phospho-Src Family Tyr529 (44-662G)	Invitrogen	Cat#44-662G
tSrc (L4A1)	Cell signaling	Cat#2110S
phospho-p44/42 MAPK (Erk1/2) Thr202/Tyr204 (D13.14.4E)	Cell signaling	Cat#4370S
p44/42 MAPK (Erk1/2) (137F5)	Cell signaling	Cat#4695S
p38 MAPK (#9212)	Cell signaling	Cat#9212S
phospho-p38 MAPK Thr180/Tyr182 (3D7)	Cell signaling	Cat#9215S
phospho-Syk Tyr525/526 (C87C1)	Cell signaling	Cat#2710S
tSyk (PA5-27262)	Invitrogen	Cat#PA5-27262
anti-rabbit IgG, HRP-linked Antibody	Cell signaling	Cat#7074
anti-mouse IgG, HRP-linked Antibody	Cell signaling	Cat#7076
**Chemicals, peptides, and recombinant proteins**		
DAPI	Sigma Aldrich	Cat#D9542
Ketamine	Sanofi Winthrop Pharmaceuticals	N/A
Xylazine	Elanco	N/A
Erythrocyte lysis buffer	BioLegend	Cat#420302
Hanks' Balanced Salt Solution (HBSS)	Sigma Aldrich	Cat#14175-053
Calcium chloride ($CaCl_2$)	Sigma	Cat#C7902-500G
Magnesium chloride ($MgCl_2$)	Roth	Cat#2189.2
Bovine serum albumin (BSA)	Serva	Cat#11930.04
Ethylenediaminetetraacetic acid (EDTA)	Sigma Aldrich	Cat# E9884-100G
Paraformaldehyde (PFA)	Sigma Aldrich	Cat#P6148-1KG

Histoplast Paraffin	Thermo Fisher	Cat#12683026
Hemalum solution acid according to Mayer	Roth	Cat#T865.2
Eosin Y Solution, Alcoholic	Sima Aldrich	Cat#HT110116-500ML
Safranin O	Sigma Aldrich	Cat#S8884-25G
Fast green	Sigma Aldrich	Cat#F7252-5G
Sucrose	Merck	Cat#573113-5KG
Polyvinylpyrrolidone (Sigma Aldrich)	Sigma Aldrich	Cat#P5288-100G
Tissue-Tek OCT compound	Sakura	Cat#4583
Isotonic sodium chloride (NaCl, 0,9%) solution	Braun	N/A
CXCL1 (recombinant, murine)	Peprotech	Cat#250-11
TNFA (recombinant, murine)	BioLegend	Cat#575206
LTB4	Cayman Chemical	Cat#Cay20110-50
Fibrinogen From Human Plasma, Alexa 647 Conjugate	Invitrogen	Cat#F35200
Percoll	Sigma Aldrich	Cat#GE17-0891-01
PBS (phosphate buffered saline)	PAN	Cat#P04-36500
Dulbecco's modified eagle's medium (DMEM)	PAN	Cat#P04-03600
Pancoll 1077	PAN	Cat#P04-60500
Pancoll 1119	PAN	Cat#P04-60150
Penicillin-streptomycin	PAN	Cat#P06-07100
WEHI-3B	purified from cell supernatant	N/A
Human serum albumin (HSA)	CSL Behring	ATC-Code: B05AA01
Fetal calf serum (FCS)	PAN	Cat#P30-3306
Superoxide dismutase (SOD)	Sigma	Cat#S5395-15Ku
Cytochrome c	Sigma Aldrich	Cat#C7752-100MG
Aprotinin	Sigma Aldrich	Cat#A6279-5ML
Dithiothreitol (DTT)	Sigma Aldrich	Cat#D0632-10G
Pefabloc SC	Sigma Aldrich	Cat#11429868001
Pepstatin A	Sigma Aldrich	Cat#P5318-5MG
Sodium fluoride	Sigma Aldrich	Cat#S7920-100G
Sodium orthovanadate	Sigma Aldrich	Cat#450243-10G
Leupeptin	Sigma	Cat#L2884-10mg
Diisopropylfluorophosphate (DFP)	Sigma Aldrich	Cat# D0879-1G
Tris (Tris-(hydroxymethyl)-aminomethan)	Roth	Cat#0188.4
Sodium chloride	Sigma	Cat#S3014-1KG
Triton-X100	Sigma	Cat#T8787-250ML
Sodium dodecyl sulfate (SDS)	Roth	Cat# 0133.3
Sodium deoxycholate	Sigma	Cat#30970
ECL Prime Western Blotting Detection Reagent	Cytiva	Cat#RPN2232
Formaldehyde	Roth	Cat#CP10.1
HEPES (4-(2-Hydroxyethyl)-piperazin-1-ethansulfonsäure)	Sigma	Cat#H0887
D-glucose	Roth	Cat#X997.2
L-glutamine	PAN	Cat#P04-80100

RPMI 1640 medium (w/o L-glutamine, w/o phenol red, w 2,0g/l $NaHCO_3$)	PAN	Cat#P04-16516
Absolute counting beads	Thermo Fisher	Cat#C36950
Amersham ECL Prime Western Blotting Detection Reagents	GE Healthcare	Cat#RPN2232
Tween 20	Sigma	Cat#P9416-100ML
2-propanol, ≥99,8 %	Roth	Cat#6752.4
Ethanol, ~96%	Sigma	Cat#02857-2.5L
Xylenes (xylene mixture of isomers)	Sigma	Cat#214736-1L
EDTA (~ 10 %) decalcifying solution	Morphisto	Cat#12584.01000
**Critical commercial assays**		
Mouse Complement Component C5a DuoSet ELISA	R&D Systems	Cat# DY2150
LTB4 Parameter Assay Kit	R&D Systems	Cat# KGE006B
Mouse IL-1 beta/IL-1F2 DuoSet ELISA	R&D Systems	Cat# DY401
Olink Target 48 Mouse Cytokine panel	Olink, Thermo Fisher	https://olink.com/products/olink-target-48-mouse
**Experimental models: organisms/strains**		
Ptprc$^{-/-}$ mice	Zhu et al. (1)	N/A
Ptprj$^{-/-}$ mice	Zhu et al. (1)	N/A
Ptprc$^{-/-}$*Ptprj*$^{-/-}$ mice	Zhu et al. (1)	N/A
**Software and algorithms**		
Adobe Illustrator (version 27.9.4)	Adobe	N/A
FlowJo (version 10.8.3)	BD Biosciences	https://www.flowjo.com/solutions/flowjo
Prism (version 9 and 10)	GraphPad	https://www.graphpad.com
ImageJ (version v.1.54h)	Wayne Rasband (formerly National Institutes of Health)	https://imagej.net/ij/
SlideBook (version 5)	Intelligent Imaging Innovations	https://www.intelligent-imaging.com/slidebook
RStudio (version 2024.09.0+375) with ggplot2 (version 3.5.1) and pheatmap (version 3.5.1) package	Posit Software, PBC	https://posit.co/download/rstudio-desktop/
R (version 4.4.1)	The R Project for Statistical Computing	https://www.R-project.org/

OsteoMeasure image analysis system	Osteometrics	N/A
Image Lab Software (version 6.1.0)	Bio-Rad	N/A
BioTek Gen5 Software for Imaging & Microscopy	Agilent	N/A
**Other**		
digital thickness gauge	Hedue	CAT#S365
XN-1000 hemacytometer	Sysmex	N/A
Microm HM340E	Thermo Fisher	N/A
Lionheart FX microscope	BioTek	N/A
Zeiss Axioskop 2 microscope	Zeiss	N/A
Cryostat CM1950	Leica	N/A
digital camera	Sensicam QE	N/A
BD FACSCantoII	BD Biosciences	N/A
Clear Strip-well Microplates (96 wells)	R&D Systems	Cat#DY990
Immulon 4HBX plates	Thermo Fisher	Cat#3855
ChemiDoc XRS+ system	Bio-Rad	N/A
Synergy Mx Plate reader	Bio-Rad	N/A
5L-Cytek Aurora	Cytek Biosciences	N/A

References

1. Zhu JW, Brdicka T, Katsumoto TR, Lin J, Weiss A. Structurally Distinct Phosphatases CD45 and CD148 Both Regulate B Cell and Macrophage Immunoreceptor Signaling. *Immunity* (2008) 28:183–196. doi: 10.1016/j.immuni.2007.11.024

Supplementary Figures

All supplementary figures (S1-S6) are shown on the following pages.

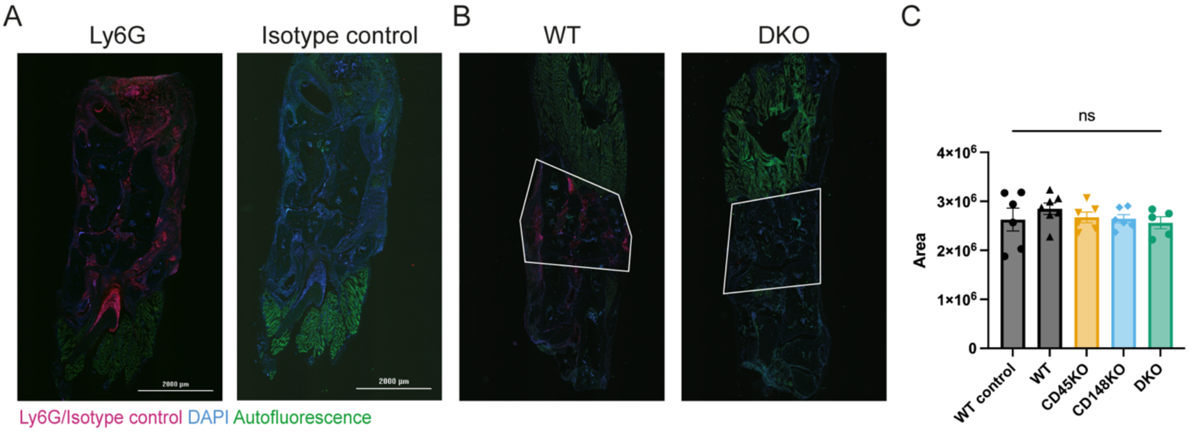

Figure S1: Immunofluorescence Ly6G staining of ankle frozen tissue sections. (A) Isotype control was included to identify nonspecific binding of secondary antibody. Representative images are shown with 4x magnification. (B) Measurement of Ly6G intensity in selected ankle areas (4x magnification) of WT and DKO mice 7 days after K/BxN serum injection. (C) Comparison of the size of ankle areas between the genotypes (n= 5-7 mice per group). All data are presented as mean +/- SEM, one-way ANOVA, ns = not significant.

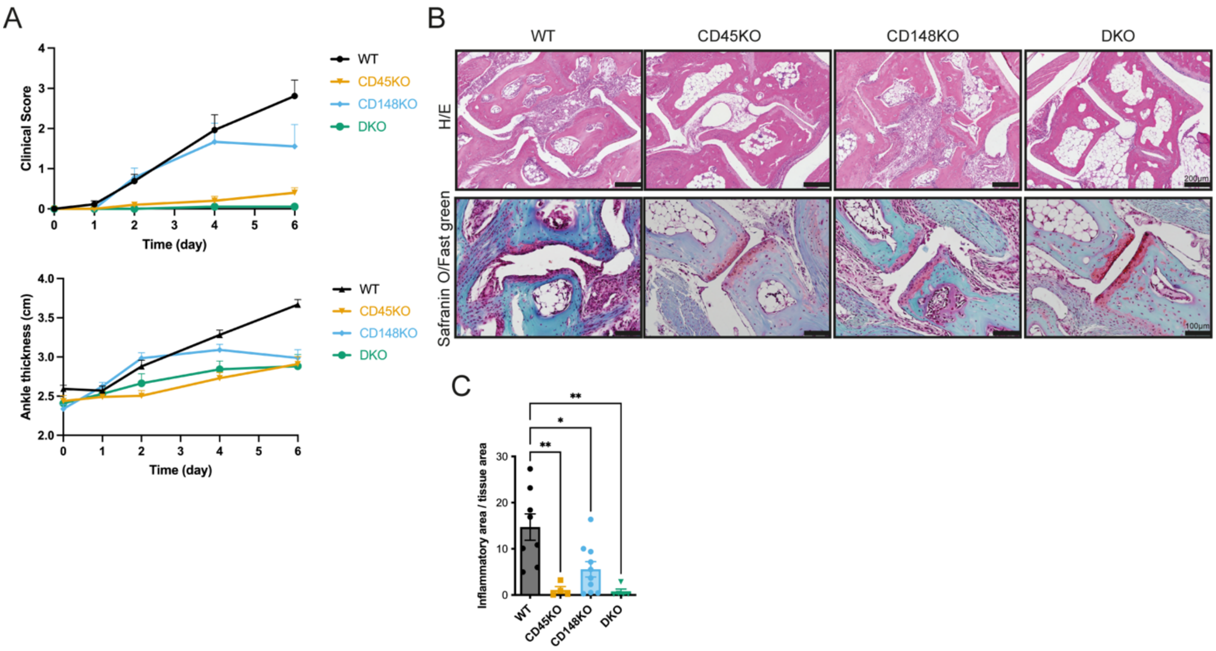

Figure S2: Hematopoietic deficiency of CD45 and CD148 partially prevents from onset and progression of serum transfer-induced arthritis. For chimeric mice, irradiated WT mice received hematopoietic stem cells from KO mice. (A) Clinical score and ankle thickness were assessed over 6 days. (B) Afterwards, mice were sacrificed and hind paws harvested, paraformaldehyde-fixed, and stained with H/E and Safranin O/Fast green. Representative images are shown. (C) Histomorphometric data of paw tissue from chimeric mice were obtained using the OsteoMeasure System (n=9-13 mice in each group). All data are presented as mean +/- SEM, one-way ANOVA, *p<0.05, **p<0.01.

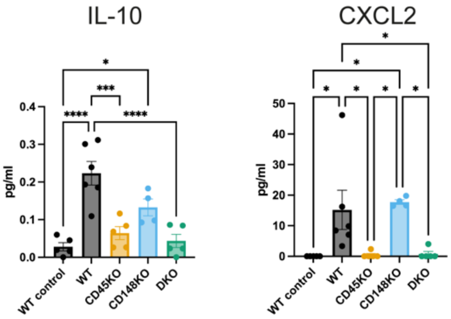

Figure S3: Concentration of IL-10 and CXCL2 in SF of WT, CD45KO, CD148KO, and DKO mice. SF was collected from the knees 7 days after K/BxN serum injection. Absolute quantification as individual graphs of IL-10 and CXCL2 concentration are shown (ROUT=2%, n=4-6 mice in each group). All data are presented as mean +/- SEM, one-way ANOVA, *$p<0.05$, ***$p<0.001$, ****$p<0.0001$.

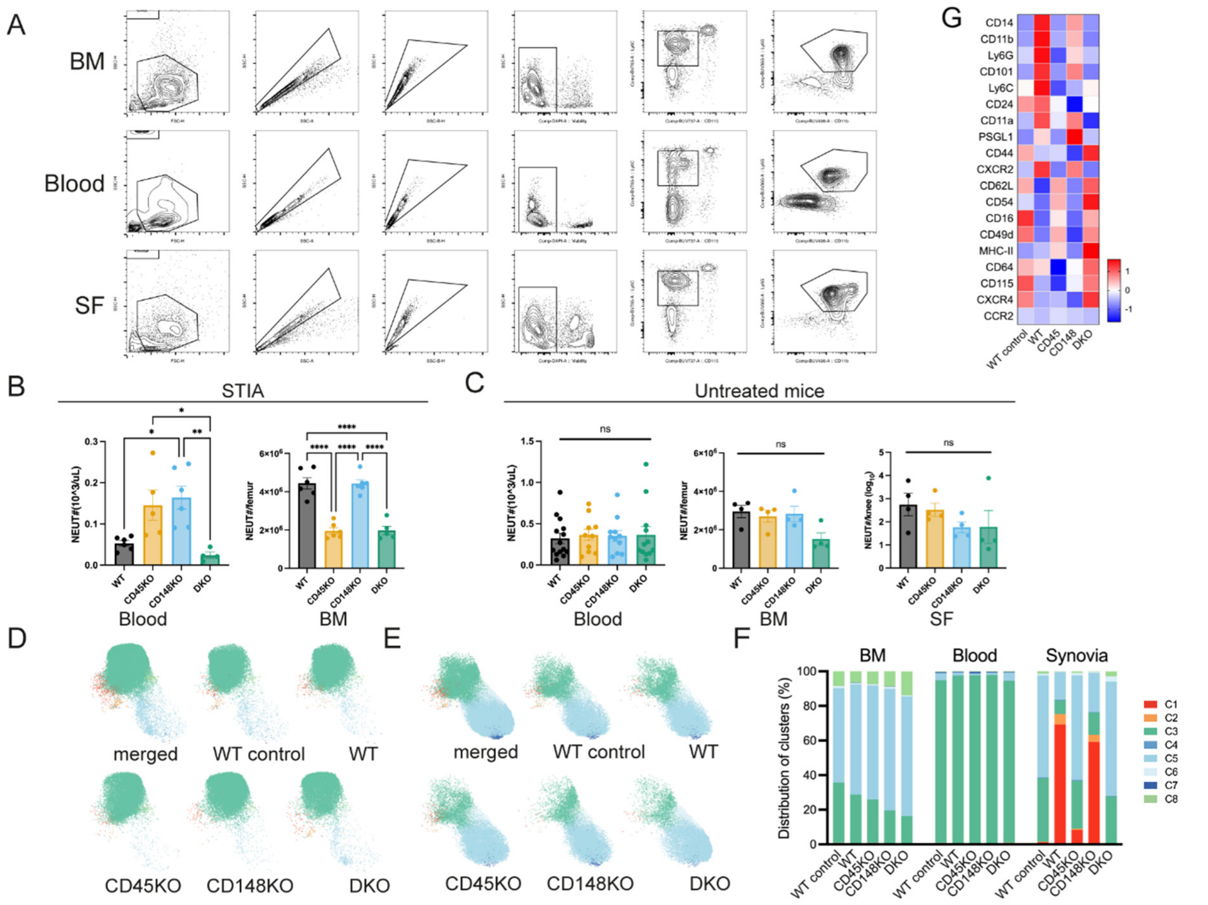

Figure S4: Gating strategy for neutrophils from BM, blood, and SF, and neutrophil counts of STIA and untreated WT, CD45KO, CD148KO, and DKO mice. (A) Illustration of the gating strategy for identification of neutrophils from BM, blood, and SF. (B) Neutrophil counts in blood and BM from each group after STIA were determined (n=4-6 mice in each group). (C) Measurement of neutrophil counts in BM, blood, and SF from untreated WT, CD45KO, CD148KO, and DKO mice (n=4-5 mice for BM and SF samples and n=10-14 mice for blood samples in each group). Blood counts were measured automatically on Sysmex multiparameter analyzer. (D-E) Based on UMAP analysis, clustering of neutrophils was performed and visualized for blood (D) and BM (E) samples according to each genotype (n=5-6 mice in each group). (F) Cluster distribution within tissues according to Figure 4D. (G) Heatmap of marker expression in SF samples according to Figure 4G. All data are presented as mean +/- SEM, one-way ANOVA, ns = not significant, *p<0.05, **p<0.01, ****p<0.0001.

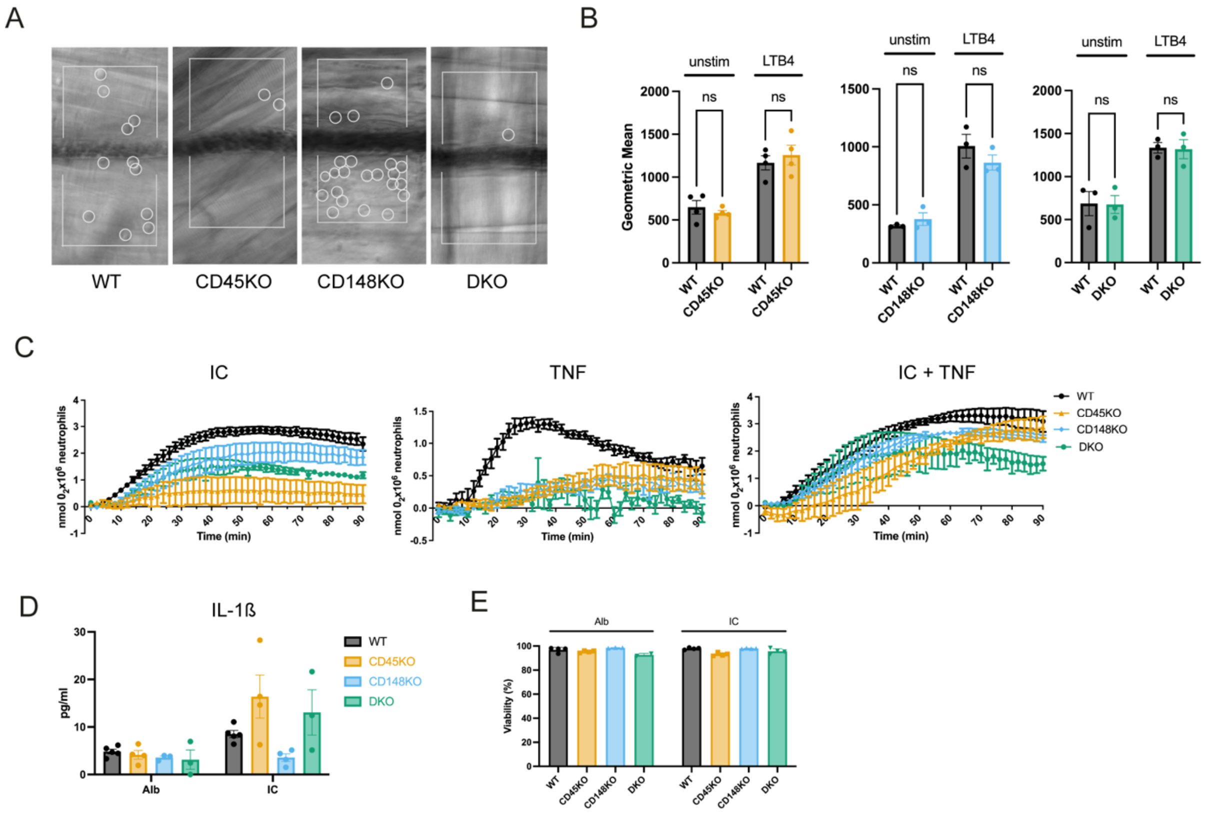

Figure S5: ICAM-binding assay, ROS production, and determination of viability during incubation with ICs. (A) Representative reflected light oblique transillumination images 2 h after TNF application. White circles represent transmigrated leukocytes within the tissue, border marking indicates an area of 75 × 100 µm. (B) Binding of the β2-integrin binding partner ICAM-1 (LFA-1, n=3-4 mice in each group) was assessed by flow cytometry after BM-derived neutrophils were stimulated with LTB4. The experiments were obtained in parallel to the stimulation with CXCL1 (Figure 4C, unstimulated data are already shown). (C) BM-derived neutrophils were plated with or without TNF on uncoated or IC-coated plates for 90 min, and ROS production was measured via cytochrome c (n=3-8 mice in each group). (D) BM-derived neutrophils were incubated on IC- or albumin-coated (Alb) plates for 6 hours, and IL-1ß was measured in the supernatants by ELISA. (E) Subsequently, BM-derived neutrophils were stained with DAPI, and viability was assessed by flow cytometry. All data are presented as mean +/- SEM, two-way ANOVA, ns = not significant.

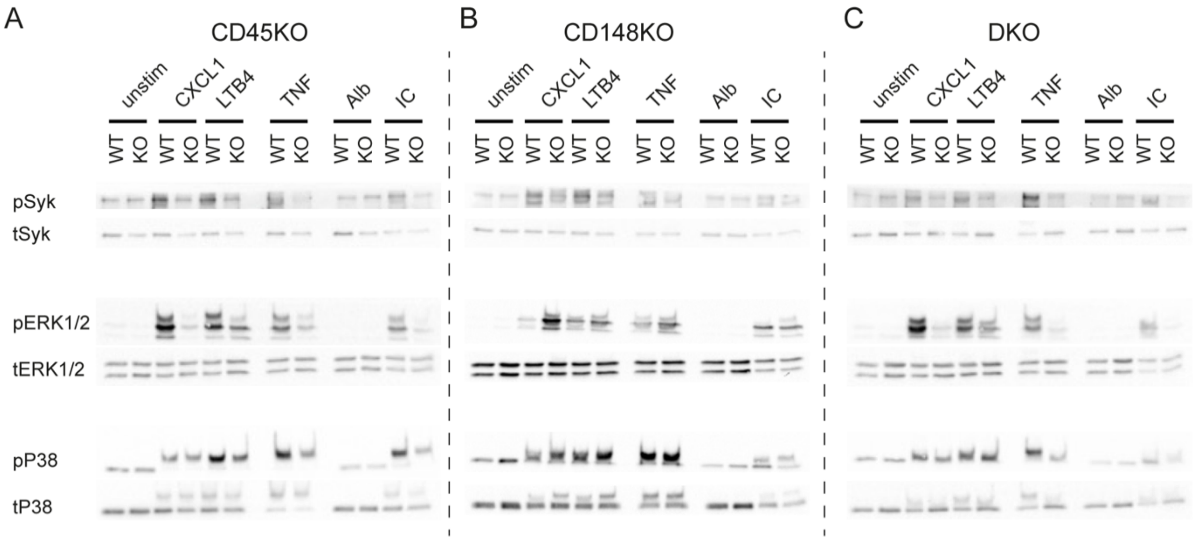

Figure S6: Representative Western blots of total lysates after GPCR- and Fc-mediated stimulation showing phosphorylation of Syk, ERK1/2, and P38. BM-derived neutrophils were left untreated or stimulated with CXCL1 or LTB4 for 2 min or TNF for 15 min. Additionally, BM-derived neutrophils were incubated on IC-plates for 15 min. Therefore, Alb-coated plates were used as controls. Cells were lysed and immunoblotted with Ab against total-Syk, phospho-Syk, total-P38, phospho-P38, total-P42/44, and phospho-P42/44. Representative Western blots of total lysates from CD45KO (A), CD148KO (B), and DKO (C) neutrophils, each compared to WT, showing the phosphorylation of Syk, ERK1/2, and P38.

Supplementary Information

ARRIVE guidelines are provided below

ARRIVE

The ARRIVE guidelines 2.0: author checklist

The ARRIVE Essential 10

These items are the basic minimum to include in a manuscript. Without this information, readers and reviewers cannot assess the reliability of the findings.

Item		Recommendation	Section/line number, or reason for not reporting
**Study design**	1	For each experiment, provide brief details of study design including:<br>a. The groups being compared, including control groups. If no control group has been used, the rationale should be stated.<br>b. The experimental unit (e.g. a single animal, litter, or cage of animals).	The groups compared are indicated in the corresponding figure legend. Control groups were included in all experiments, usually referred to as WT control or WT unstimulated (unstim). The experimental unit for each experiment is described in the corresponding figure legends.
**Sample size**	2	a. Specify the exact number of experimental units allocated to each group, and the total number in each experiment. Also indicate the total number of animals used.<br>b. Explain how the sample size was decided. Provide details of any *a priori* sample size calculation, if done.	The exact number of experimental units is specified in the figure and figure legends. Sample size was decided based on previously published and unpublished data.
**Inclusion and exclusion criteria**	3	a. Describe any criteria used for including and excluding animals (or experimental units) during the experiment, and data points during the analysis. Specify if these criteria were established *a priori*. If no criteria were set, state this explicitly.<br>b. For each experimental group, report any animals, experimental units or data points not included in the analysis and explain why. If there were no exclusions, state so.<br>c. For each analysis, report the exact value of *n* in each experimental group.	Animals were only excluded if there was an error during the experiments, e.g. during the tissue processing. If indicated, a ROUT outlier test (Q=2%) was performed. Exact values of n in each experimental group are described in the figure legend.
**Randomisation**	4	a. State whether randomisation was used to allocate experimental units to control and treatment groups. If done, provide the method used to generate the randomisation sequence.<br>b. Describe the strategy used to minimise potential confounders such as the order of treatments and measurements, or animal/cage location. If confounders were not controlled, state this explicitly.	WT animals were randomly divided into two groups receiving NaCl or K/BxN serum injection. For STIA model, test time was between 08.30 am to 12.30 pm daily, testing order was randomized daily.
**Blinding**	5	Describe who was aware of the group allocation at the different stages of the experiment (during the allocation, the conduct of the experiment, the outcome assessment, and the data analysis).	During the STIA experimental setup, investigators were blinded for genotype of the mice.

**Outcome measures**	6	a. Clearly define all outcome measures assessed (e.g. cell death, molecular markers,or behavioural changes).<br>b. For hypothesis-testing studies, specify the primary outcome measure, i.e. the outcome measure that was used to determine the sample size.	The following parameters were assessed: Clinical scoring, Ankle thickness, Histological analyses, Neutrophil infiltration and Chemokine/cytokine profile in the joints
**Statistical methods**	7	a. Provide details of the statistical methods used for each analysis, including software used.<br>b. Describe any methods used to assess whether the data met the assumptions of the statistical approach, and what was done if the assumptions were not met.	All statistical methods and software used are described in the Material and Methods section and indicated in the corresponding figure legend.
**Experimental animals**	8	a. Provide species-appropriate details of the animals used, including species, strain and substrain, sex, age or developmental stage, and, if relevant, weight.<br>b. Provide further relevant information on the provenance of animals, health/immune status, genetic modification status, genotype, and any previous procedures.	All species-appropriate details including housing conditions, name of the ethical review committee that has approved the use of animals in this study and any protocol numbers are described in the Material and Methods section as well as in the Supplemental material.
**Experimental procedures**	9	For each experimental group, including controls, describe the procedures in enough detail to allow others to replicate them, including:<br>a. What was done, how it was done and what was used.<br>b. When and how often.<br>c. Where (including detail of any acclimatisation periods).<br>d. Why (provide rationale for procedures).	The procedures are described in detail in the Materials and methods section and in the supplementary material.
**Results**	10	For each experiment conducted, including independent replications, report:<br>a. Summary/descriptive statistics for each experimental group, with a measure of variability where applicable (e.g. mean and SD, or median and range).<br>b. If applicable, the effect size with a confidence interval.	For each experiment conducted, mean +/- SEM, used statistical test and p-values are indicated in the figure and corresponding figure legend.
